# Correction: A simple and straightforward synthesis of phenyl isothiocyanates, symmetrical and unsymmetrical thioureas under ball milling

**DOI:** 10.1039/d1ra90128a

**Published:** 2021-07-05

**Authors:** Ze Zhang, Hao-Hao Wu, Ya-Jun Tan

**Affiliations:** School of Biological and Chemical Engineering, Anhui Polytechnic University Wuhu 241000 China zhangze@ustc.edu.cn +86 553 2871254

## Abstract

Correction for ‘A simple and straightforward synthesis of phenyl isothiocyanates, symmetrical and unsymmetrical thioureas under ball milling’ by Ze Zhang *et al.*, *RSC Adv.*, 2013, **3**, 16940–16944, DOI: 10.1039/C3RA43252A.

The authors regret that it was not clear in the original article that part of the graphical abstract image had been reproduced from the graphical abstract of an earlier *ChemComm* paper.^1^ Although the *ChemComm* article was cited as ref. 15*b*, and here as ref. 1, it was not made clear that some of the image was reproduced from this article.

The Royal Society of Chemistry apologises for these errors and any consequent inconvenience to authors and readers.

## Supplementary Material
